# Annexin A2-Mediated Plasminogen Activation in Endothelial Cells Contributes to the Proangiogenic Effect of Adenosine A_2A_ Receptors

**DOI:** 10.3389/fphar.2021.654104

**Published:** 2021-04-27

**Authors:** María D. Valls, María Soldado, Jorge Arasa, Miguel Perez-Aso, Adrienne J. Williams, Bruce N. Cronstein, M. Antonia Noguera, M. Carmen Terencio, M. Carmen Montesinos

**Affiliations:** ^1^ Departament of Pharmacology, Faculty of Pharmacy, Universitat de València, Valencia, Spain; ^2^ Instituto Interuniversitario de Investigación de Reconocimiento Molecular y Desarrollo Tecnológico (IDM), Universitat Politècnica de València, Universitat de València, Valencia, Spain; ^3^ Division of Translational Medicine, Department of Medicine, NYU School of Medicine, New York, NY, United States; ^4^ Division of Rheumatology, Department of Medicine, NYU School of Medicine, New York, NY, United States; ^5^ Medical Science Building, NYU Langone Health, New York, NY, United States; ^6^ Instituto Universitario de Biotecnología y Biomedicina (BIOTECMED) Universitat de València, Valencia, Spain

**Keywords:** annexin A2, microvascular endothelial cells, plasminogen activator inhibitor-1, tissue plasminogen activator, urokinase plasminogen activator, adenosine receptors

## Abstract

Adenosine A_2A_ receptor mediates the promotion of wound healing and revascularization of injured tissue, in healthy and animals with impaired wound healing, through a mechanism depending upon tissue plasminogen activator (tPA), a component of the fibrinolytic system. In order to evaluate the contribution of plasmin generation in the proangiogenic effect of adenosine A_2A_ receptor activation, we determined the expression and secretion of t-PA, urokinase plasminogen activator (uPA), plasminogen activator inhibitor-1 (PAI-1) and annexin A2 by human dermal microvascular endothelial cells stimulated by the selective agonist CGS-21680. The plasmin generation was assayed through an enzymatic assay and the proangiogenic effect was studied using an endothelial tube formation assay in Matrigel. Adenosine A_2A_ receptor activation in endothelial cells diminished the release of PAI-1 and promoted the production of annexin A2, which acts as a cell membrane co-receptor for plasminogen and its activator tPA. Annexin A2 mediated the increased cell membrane-associated plasmin generation in adenosine A_2A_ receptor agonist treated human dermal microvascular endothelial cells and is required for tube formation in an *in vitro* model of angiogenesis. These results suggest a novel mechanism by which adenosine A_2A_ receptor activation promotes angiogenesis: increased endothelial expression of annexin A2, which, in turn, promotes fibrinolysis by binding tPA and plasminogen to the cell surface.

## Introduction

Adenosine is a ubiquitous nucleoside that participates actively in regulating different physiological processes involved in tissue repair and wound healing, inflammatory and immune responses, or formation of new blood vessels (Valls et al., 2009; Borea et al., 2018). Among the four distinct extracellular G protein-coupled adenosine receptors (ARs) named A_1_, A_2A_, A_2B_ and A_3_, that mediate its effects, A_2A_ receptor activation has been reported to promote wound healing (Shaikh and Cronstein, 2016; Colangelo et al., 2020; Troncoso et al., 2020). The contribution of adenosine A_2A_ receptor in wound healing has been further confirmed in A_2A_-deficient mice, characterized by reduced blood vessel network and delayed granulation tissue formation (Montesinos et al., 2002). Furthermore, the adenosine A_2A_ receptor agonist CGS-21680 stimulates both vasculogenesis and angiogenesis at an early stage (<3 days) of wound repair (Montesinos et al., 2004).

Plasminogen and its active form plasmin belong to an enzymatic system involved in intravascular and extravascular fibrinolysis, as well as in tissue repair and remodeling by regulating extracellular matrix degradation, cell migration, tissue formation, angiogenesis, and embryogenesis (Rubina et al., 2017; Plekhanova et al., 2019). The activity of this system is self-regulated by several factors such as tissue-type plasminogen activator (t-PA), urokinase-type plasminogen activator (u-PA) or the plasminogen activator principal inhibitor PAI-1 (Simone et al., 2015). In addition, there are other molecules that interact with the fore mentioned plasminogen factors resulting in the modulation of their activity. Annexin A2 is a member of a family of calcium-dependent membrane-binding proteins that acts as co-receptor for plasminogen and t-PA (Liu and Hajjar, 2016; Mirsaeidi et al., 2016). Consequently, annexin A2 deficient mice present deficiencies in plasmin generation through a mechanism dependent on t-PA (Ling et al., 2004).

Our previous results confirm that adenosine A_2A_ receptor activation promotes wound closure by a mechanism that depends on t-PA, but not on u-PA expression (Montesinos et al., 2015). We also found that increased revascularization of the wound bed upon treatment with agonists of adenosine A_2A_ receptors occurs via a mechanism dependent on t-PA (Montesinos et al., 2015). In this study, we sought to provide insight into the pharmacological promotion of angiogenesis by an adenosine receptor agonist through a mechanism dependent upon plasminogen activation. We showed that adenosine A_2A_ receptor activation diminishes PAI-1 production and promotes tubular network formation in human dermal microvascular endothelial cells (HDMVEC). This proangiogenic effect observed upon adenosine A_2A_ receptor activation is supported by the upregulation of annexin A2 associated with t-PA.

## Materials and Methods

### Materials

The adenosine 2A receptor agonist CGS-21680 (2-p-[2-carboxyethyl] phenethyl-amino-5′-N-ethylcarboxamido-adenosine) was obtained from Tocris bioscience (Cat #1063). Polyclonal goat antibodies against tPA (Cat #387), uPA (Cat #398) and PAI-1 (Cat 395G) were obtained from American Diagnostica inc.; monoclonal mouse antibody against annexin A2 from BD Transduction Laboratories (Cat #610069); rabbit antibody against *ß*-actin from Sigma Chemical Co. (Cat #A2066); Alexa fluor^®^ 488 goat anti-mouse (Cat #A1101) antibody from Molecular Probes Ltd. All other materials were the highest quality that could be obtained.

### Cell Culture

Human dermal microvascular endothelial cells (HDMVEC) from neonate foreskin (Lonza, Cat #CC-2505) were cultured up to 80–90% confluence in supplemented EGM-2MV medium (Lonza, Cat #CC-3202) at 37°C and 5% CO_2_ in a humidified atmosphere. Cells used in all experiments were between passages 5 and 7. The night prior the experiments, medium was replaced to 0.1% Bovine Serum Albumin (BSA, ELISA grade, Sigma Chemical Co., Cat #A7030) supplemented EBM-2 medium (Lonza, Cat #CC-3156). All experiments were carried out in 0.1% BSA basal medium.

### ELISA

HDMVEC, cultured in 24 well plates, were incubated in the absence (control) or presence of increasing concentrations (10^−7^–10^–5^ M) of the selective A_2A_ adenosine receptor agonist CGS-21680 in 0.1% BSA basal medium for 24 h at 37 °C and 5% CO_2_. Supernatants were collected and frozen at -80 °C until ELISA determination. Cell viability was determined by cellular reduction of 3-(4,5-dimethylthiazol-2-yl)-2,5-diphenyltetrazolium bromide (MTT, 0.2 mg/ml, Sigma Chemical Co. Cat #M2003) to formazan, solubilization in DMSO and absorbance measurement at 490 nm. Concentration of total tPA, uPA and PAI-1 antigens present in conditioned media were determined using commercially available assays: IMUBIND^®^ tPA (Cat #860), uPA (Cat #894) or PAI-1 (Cat #822) ELISA (American Diagnostica Inc.). Sensitivity of the kit for tPA is 0.2 ng/ml, for uPA is 0.02 ng/ml and for PAI-1 is 1 ng/ml. Results were normalized to 10^5^ viable cells per well.

### Real Time RT-PCR

Total RNA (1 µg) isolated from cultured cells using Trizol reagent (Invitrogen Cat# 15596018) was transcribed into cDNA (RT) using the RNA PCR Core Kit (Applied Biosystems™ N8080143). Aliquots of RT were subjected to real-time PCR using the Mx3005P system (Stratagene, La Jolla, CA), SYBR-Green Brilliant Master Mix (Stratagene Cat #600548) and specific primers for t-PA (Forward 5′-CCC​AGA​TCG​AGA​CTC​AAA​GC-3′ Reverse 5′-TGG​GGT​TCT​GTG​CTG​TGT​AA-3′, NCBI Reference Sequences: NM_000921 and NM_033011), u-PA (Forward 5′-ATT​CAC​CAC​CAT​CGA​GAA​CC-3′ Reverse 5′-TCC​ACC​TCA​AAC​TTC​ATC​TCC-3′, NCBI Reference Sequence: NM_002658), PAI-1 (Forward 5′-CTG​GTT​CTG​CCC​AAG​TTC​TC–3′ Reverse 5′-GAC​TGT​TCC​TGT​GGG​GTT​GT-3′, NCBI Reference Sequence: NM_000602), and GAPDH (Forward 5′-AAC​ATC​ATC​CCT​GCC​TCT​AC–3′ Reverse 5′-CCC​TGT​TGC​TGT​AGC​CAA​AT-3′); Annealing Temperature: 60°C. All values were normalized to GAPDH as described (Che et al., 2007). All primers were designed in a way that the PCR products spanned an intron.

### Cell Immunofluorescence Microscopy

HDMVEC were grown on chamber slides (Nalge Nunc International, Cat #177402). After treatment, medium was discarded, and cells were fixed in ice cold 70% ethanol and blocked with 2% BSA in 0.01% (vol/vol) Tween 20 PBS - (PBST). Slides were incubated with primary antibodies against proteins of interest and subsequently incubated with Alexa Fluor 488 secondary antibodies, nuclei were counterstained with Hoerschst 33342 (Molecular Probes Inc. Cat #H21492) and slides were mounted in fluorescence mounting medium (DakoCytomaton, Glostrup, Denmark). Negative controls were run in parallel without the primary antibody. Fluorescence was detected with a Nikon Eclipse e800 microscope coupled with an automated imager Nikon ACT-1 (Nikon Instruments Inc. Melville, NY) and fluorescence intensity was blindly quantified with MetaMorph software (Molecular Devices Corporation, Downington, PA) by an independent observer.

### Confocal Laser Scanning Microscopy

HDMVEC were seeded on poly-l-lysine (100 μg/ml) coated 24 mm diameter glass coverslips in six-well plates. After treatment, calcium-dependent translocation of annexin A2 to the membrane (Monastyrskaya et al., 2007) was induced by addition of 10^−6^ M ionophore A23187 (Sigma Chemical Co., Cat #C7522) for 5min. Then, medium was discarded and cells were fixed in ice cold 70% ethanol, blocked with 2% BSA in 0.01% (vol/vol) Tween 20 PBS - (PBST), incubated with primary antibodies against annexin A2, subsequently incubated with Alexa Fluor 488 goat anti-mouse antibody and nuclei were counterstained with Hoerschst 33342. Confocal images were obtained on a LEICA TCS SP2 (DM-IRB) laser-scanning microscope. A z-series of images were acquired at 2 µm steps to produce individual z-stacks. The resulting images were overlaid and analyzed using the Leica software, v2.61 (Leica Geosystems AG, Heerbrugg, Suiza).

### Western Blot Analysis

HMVEC, cultured in 6 well plates, were incubated in the absence (control) or presence of the selective A_2A_ adenosine receptor agonist CGS-21680 in 0.1% BSA basal medium for 24 h at 37°C and 5% CO_2_. Supernatants were removed and cells were rinsed in ice-cold phosphate-buffered saline (PBS), pH 7.4 and lyzed at 4°C in lysis buffer (50 mM Tris pH 7.4, 150 mM NaCl, 0.1% SDS, 1% Nonidet P-40 and 0.5% sodium deoxycolate) plus protease inhibitors (leupeptin, aprotinin and PMSF). Following centrifugation (10000*g*, 10 min) equal amounts of protein (5 or 25 µg/lane) were separated by 10% SDS-PAGE under reducing conditions and electrophoretically transferred onto poly-(vinylidene difluoride) (PVDF) membranes, blocked with 3% nonfat milk in 0.1% (vol/vol) Tween 20 phosphate buffered saline (PBST) and then incubated with specific antibodies against the protein of interest. After extensive washes, blots were incubated with a horseradish peroxidase-conjugated secondary antibody and the immunoreactive bands were visualized by enhanced chemiluminescence (Amersham Biosciences, Cat #RPN2232) using the AutoChemi image analyzer and Labworks 4.6 software (UVP, Inc. Upland, CA).

### Co-Immunoprecipitation

After treatment, HDMVEC cultured in T25 flasks were lyzed with 1 ml of lysis buffer (50 mM Tris pH 7.4, 150 mM NaCl, 0.1% SDS, 1% Nonidet P-40 and 0.5% sodium deoxycolate) containing protease inhibitors (leupeptin, aprotinin and PMSF). Whole cell lysates were precleared for 30 min at 4°C with 0.25 µg of appropriate control (normal) IgG, corresponding to the host species of the primary antibody (Mouse IgG, Sigma Chemical Co., Cat #I5381), and 20 µL of protein A/G-agarose (Santa Cruz Biotechnology, Inc. Cat #sc-2003). The precleared cell lysates were subsequently incubated with 2 µg of the monoclonal antibody against annexin A2 for 2 h at 4°C before the addition of 20 µL of protein A/G-agarose, and the incubation was continued overnight at 4°C with constant rotation. Next, the agarose beads were pelleted by centrifugation at 2500 rpm (1,000*g*) for 5 min at 4°C. After extensive washes with ice-cold lysis buffer, beads were resuspended in sample loading buffer, boiled for 4–5 min, and subject to Western blotting with specific antibody against tPA.

### Plasmin Generation Test

Plasminogen activation was determined as the ability of generated plasmin to degrade a specific fluorogenic substrate by membrane bound exogenous tPA (Diaz et al., 2004). HDMVEC (10^4^ cells/well) were cultured in 96 well microplate, black, clear bottom (Corning Incorporated, Cat #CLS3603). After treatment, supernatants were removed, and cells were washed twice with PBS and incubated with 10 nM of single-chain recombinant tPA (American Diagnostica inc. Cat #173) in PBS-BSA 2% for 30 min. After washing three times with PBS to remove unbound tPA, 100 nM Glu-plasminogen (Calbiochem, manufactured by EMD Biosciencies, Inc.; Cat #528180) and 50 µM of the fluorogenic substrate D-Ala-Leu-Lys-7-amido-4-methylcoumarin (Sigma Chemical Co., Cat #A8171) in buffer 0.05 M Tris-HCl, 0.1 M NaCl, 0.01% Tween 20, pH 7,4 were added. Plasmin formation was monitored at 360-nm excitation/460-nm emission setting in a spectrofluorimeter plate reader (Perkin-Elmer WALLAC Victor^2^ 1420, Perkin-Elmer life sciencies, Turku, Finland) at 5- or 30-min intervals for 6 h at 37°C. The basal fluorescence obtained at 0 min was subtracted from each time point. Additionally, we performed a control chemical reaction without cells to monitor the kinetics of the reaction. To determine the role of plasminogen binding to HDMVEC, these assays were also performed including 6-aminocaproic acid (6-ACA, Sigma Chemical Co., Cat #A2504; 1 mM) in the final reaction mixture as antagonist of this binding.

### *In vitro* Angiogenesis Assay

200 µL of HDMVECs cell suspension (5 × 10^4^ cells/ml from passage 4) in EGM-2MV medium were seeded on 50 µL of polymerized (37°C for 30 min) Matrigel^®^ (BD Biosciences, Cat #354234) in a 96 well plate. After 18 h incubation (37°C and 5% CO_2_), Fluorophore/calcein AM (Molecular Probes, Inc. Cat #C1430) was added to stain cells. Image acquisition of tubular network was achieved by fluorescence microscopy (Nikon Eclipse e800 microscope coupled with an automated imager Nikon ACT-1, Nikon Instruments Inc. Melville, NY) and Sigma Scan Pro software (SPSS, Chicago, IL) was used to determine tube surface area (Perez-Aso et al., 2014; Vicente et al., 2016).

### Statistical Analysis

Differences between groups in the *in vitro* studies were analyzed by means of one-way analysis of variance (ANOVA) by Dunnet’s multiple comparison test performed by GraphPad Prism 4 software (GraphPad Software, Inc. San Diego, CA).

## Results

### An Adenosine A_2A_ Receptor Agonist Diminishes PAI-1 Production Without Modifying tPA and uPA Production in Human Dermal Microvascular Endothelial Cells

The expression of the key components of the plasminogen activation system, uPA and its receptor (uPAR), as well as its inhibitor PAI-1, is induced early during re-epithelialization in the migrating epithelial sheet in incisional wounds in mice (Sulniute et al., 2016). In contrast, the vascular endothelium is considered to be the major site of synthesis and release of tPA (Emeis et al., 1997). Since we have previously shown that adenosine A_2A_ receptor activation promoted wound revascularization by promoting both angiogenesis and vasculogenesis (Montesinos and Valls, 2010), we investigated its effect on human dermal microvascular endothelial cells (HDMVEC).

**FIGURE 1:**
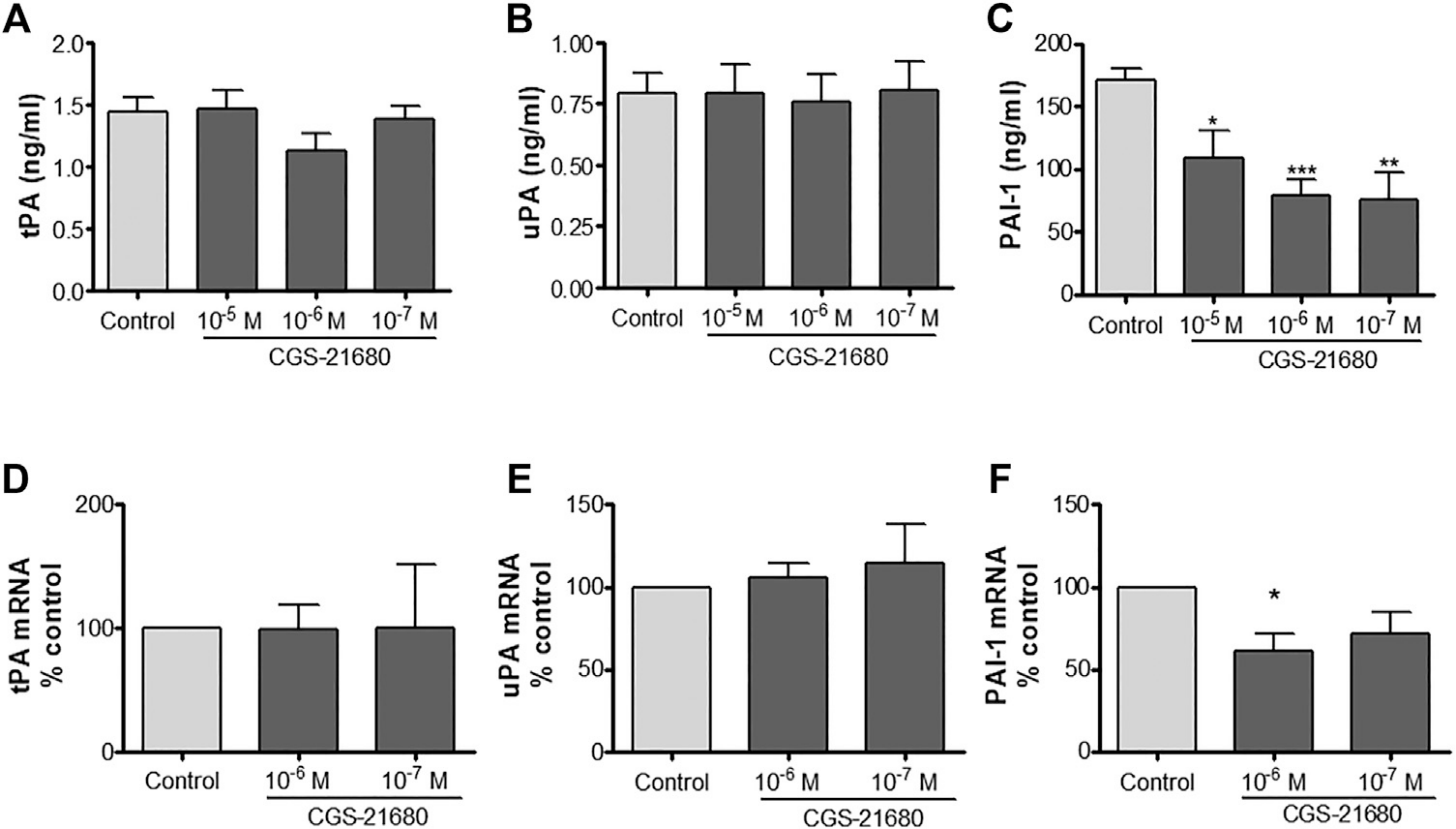
uPA, tPA and PAI-1 production by microvascular endothelial cells: HDMVEC (80–90% confluence) were incubated in the absence (control, vehicle treated) or presence of the selective adenosine A_2A_ receptor CGS-21680 for 24 h for protein detection or 4 h for mRNA expression. Supernatants were collected and frozen at −80°C until used and cells were lyzed in Trizol solution for total RNA extraction. Total tPA **(A)**, uPA **(B)**, and PAI-1 **(C)** content in supernatants were determined by ELISA. Results were normalized to 10^5^ cells. Data are presented as the mean ± SEM of four experiments in triplicate (*n* = 4). mRNA expression of tPA **(D)**, uPA **(E)**, and PAI-1 **(F)** were assessed by real-time PCR and normalized to GAPDH as described. Results are expressed as mean of percentage of control ± SEM, of 3 experiments in duplicates (*n* = 3). *p < 0.05, **p < 0.01, ***p < 0.001 vs. control, ANOVA followed by Dunnet’s postest.

HDMVEC secreted both types of plasminogen activators, tPA and uPA, in lesser amounts than their inhibitor PAI-1 (Figures 1A–C). The addition of increasing amounts of the selective A_2A_ receptor agonist did not produce any appreciable change in the secretion of tPA and uPA, but significantly diminished the levels of PAI-1 present in the conditioned media of HDMVEC (79.3 ± 12.1 ng/ml in 10^−6^ M CGS-21680 treated vs. 171.2 ± 9.3 ng/ml in untreated HDVECs, ***p < 0.001, *n* = 4, Figure 1C). Real time RT-PCR determination of the mRNA levels for tPA, uPA and PAI-1 showed that A_2A_ adenosine receptor activation did not affect tPA and uPA mRNA expression in HDMVECs while decreasing PAI-1 message levels (39% reduction from control by 10^–6^ M CGS-21680, *p* = 0.050, *n* = 3, Figures 1D–F). Therefore, the effect of adenosine A_2A_ receptor activation on the level of uPA, tPA and PAI-1 expression correlated with the effect on released proteins to the condition media.

### Adenosine A_2A_ Receptor Activation Increases Annexin A2 Expression in HDMVEC

**FIGURE 2:**
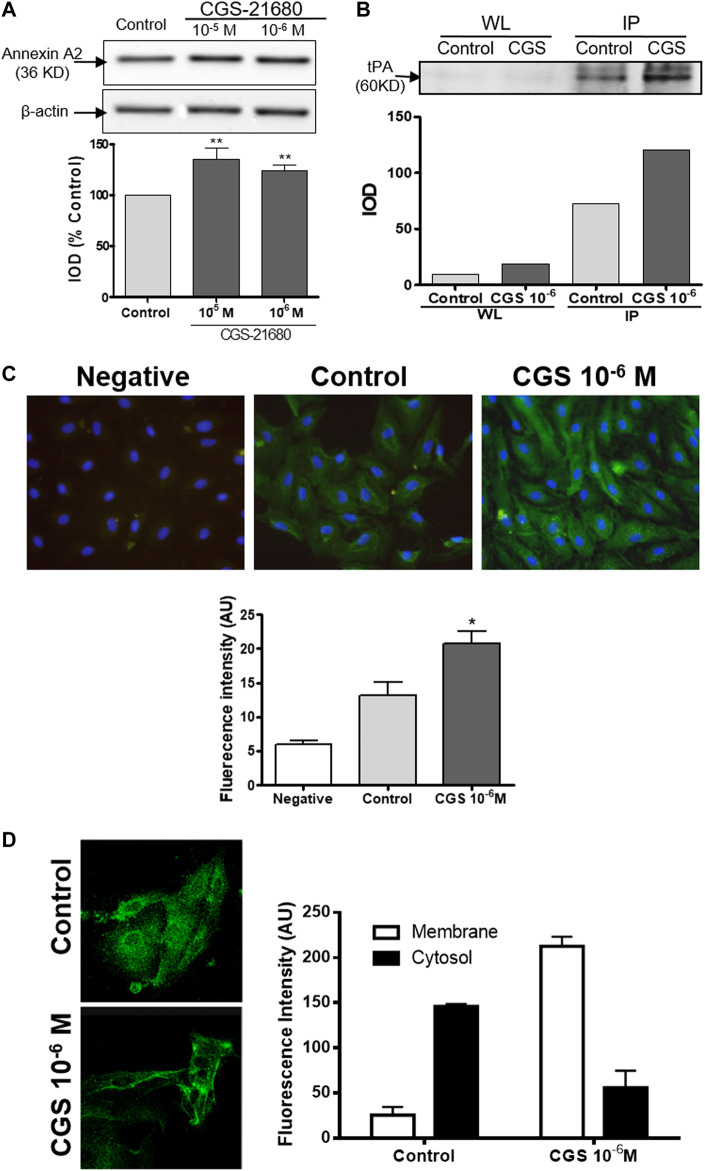
Annexin A2 production by microvascular endothelial cells: HDMVEC (80–90% confluence) were incubated in the absence (control, vehicle treated) or presence of the selective A_2A_ adenosine receptor CGS-21680 (10^−6^ M) for 24 h. **(A)** Whole cell lysates (5 μg/lane) were separated by 10% SDS-PAGE and annexin A2 and *ß*-actin were detected by immunoblotting. Protein expression was measured by optical densitometry of the bands and results were expressed as % integrated optical density (IOD) respect control and normalized to *ß*-actin and are presented as the mean ± SEM (*n* = 16). **p < 0.01 vs. control, t de Dunnet. **(B)** Co-immunoprecipitation of tPA and Annexin A2. Precleared cell lysates were immunoprecipitated with a monoclonal antibody against annexin A2, resuspended in sample loading buffer and tPA was detected by immunoblotting. Shown is a representative experiment of four. WL: Whole lysate; IP: immunoprecipitates. **(C)** ethanol fixed cells were immunostained with a monoclonal antibody against annexin A2 and a secondary antibody Alexa Fluor 488-conjugated goat antimouse, counterstained with Hoechst 33342 and visualized by fluorescence microscopy (original magnification ×400). Average fluorescence intensity of annexin A2 immunostained HDMVEC was determined with MetaMorph^®^ software. Results are expressed in intensity arbitrary units for 30 cells per condition and presented as the mean ± SEM of 5 experiments in triplicate (*n* = 5). *p < 0.05 vs. control, ANOVA followed by Dunnet’s postest. Negative: negative control without primary antibody. **(D)** A z-series of images were acquired at 2 µm steps by confocal laser scanning microscopy of ethanol fixed cells immunostained with a monoclonal antibody against annexin A2 and a secondary antibody Alexa Fluor 488-conjugated goat antimouse and counterstained with Hoechst 33342. The resulting images were overlaid and analyzed using the Leica software, v2.61. Results are expressed in intensity arbitrary units per condition and presented as the mean ± STDV (*n* = 2).

Since we had observed that CGS-21680 treatment did not affect the expression and the release of tPA by HDMVEC, we decided to investigate the production of annexin A2, a protein that acts as an endothelial cell membrane co-receptor of plasminogen and tPA (Hajjar et al., 1994). Annexin A2 is an abundant protein (∼36 KD) in HDMVECs with mainly perinuclear localization. The activation of A_2A_ adenosine receptors by CGS-21680 significantly increased the overall levels of this protein, shifting its localization toward the cellular membrane (Figures 2A,C). These findings were further confirmed by confocal laser scanning microscopy (Figure 2D).

In order to determine whether there is any physical interaction between endogenous tPA and annexin A2, we immunoprecipitated annexin A2 and observed that the amount of tPA (∼60 KD) that co-precipitated with annexin A2 increased markedly in the CGS21680-treated cells (Figure 2B).

### Increased Annexin A2 Production Contributes to Cell Associated-tPA Plasmin Generation by Adenosine A_2A_ Receptor Stimulated HDMVEC

**FIGURE 3:**
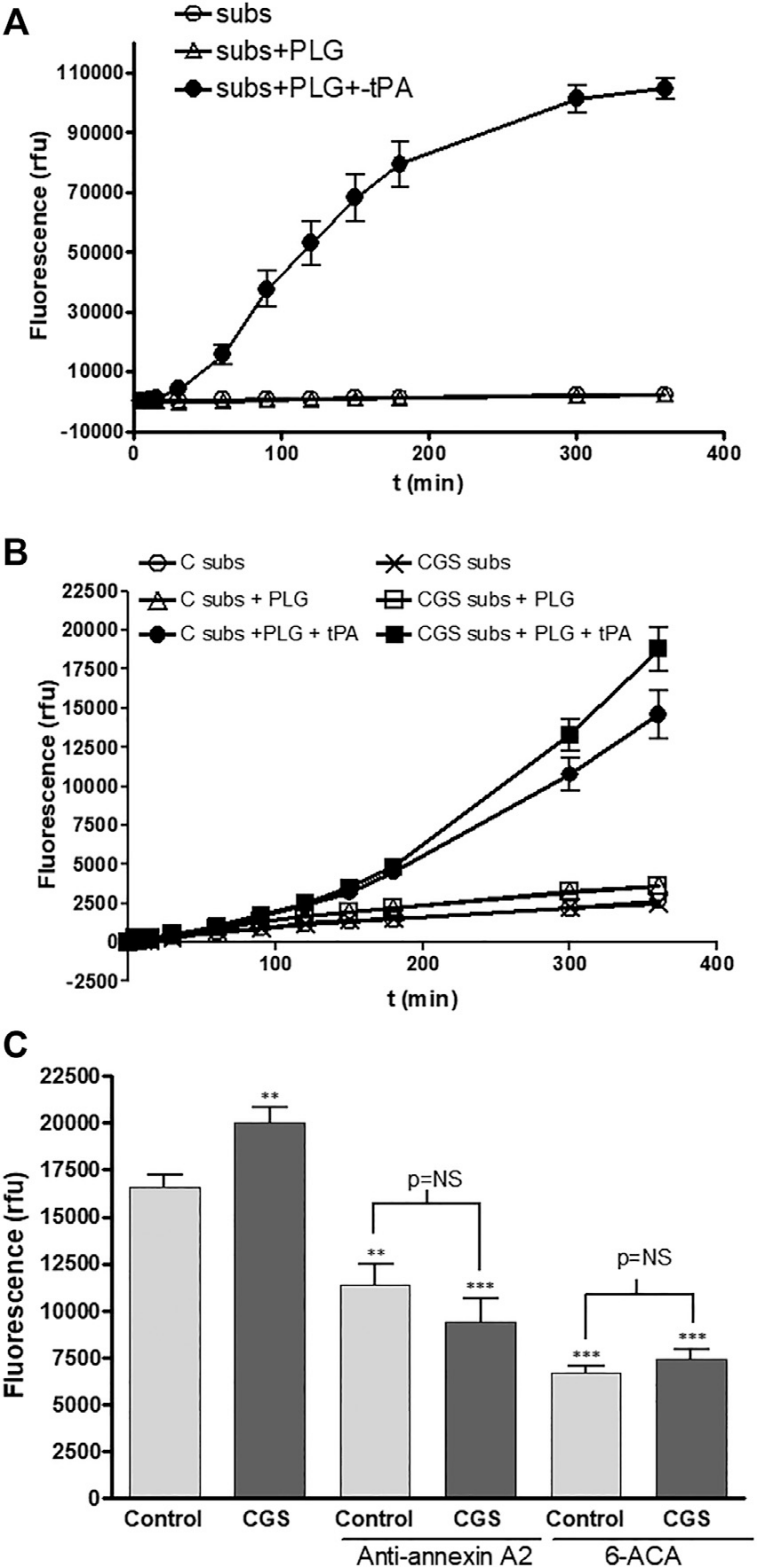
Plasminogen activation by microvascular endothelial cells: **(A)** Chemical control reaction reagents were added to wells with no cells. Fluorescence was detected at 360-nm excitation/460-nm emission setting over time. Subs: fluorogenic substrate (50 mM), PLG: Glu-plasminogen (100 nM), rtPA: recombinant tPA (10 nM). **(B)** HDMVEC (80–90% confluence) were incubated in the absence (control, vehicle treated) or presence of the selective A_2A_ adenosine receptor CGS-21680 (10^−6^ M) for 24 h. Time-course plasmin generation by cell bound-tPA was determined in cells after washing twice with PBS and incubation with 10 nM rtPA for 20 min. After two PBS washes, Glu-plasminogen (100 nM) and the substrate (50 µM) were added and fluorescence was detected. C: control vehicle-treated cells; CGS: 10^−6^ M CGS-21680-treated cells. **(C)** In other set of experiments for plasmin generation by cell bound-tPA after 6 h, monoclonal antibody anti-annexin A2 (4 μg/ml) was added 10 min prior the addition of rtPA and 6-ACA (1 mM) was incubated for 10 min prior addition of the Glu-plasminogen and the substrate. Results are expressed as mean relative fluorescence units (rfu) + SEM (*n* = 6 in quadruplicate). **p < 0.01, ***p < 0.001 vs. control cells, ANOVA followed by Dunnet’s postest.

We determined the functionality of the increased annexin A2 production by an indirect test of plasminogen activation, through the degradation of a specific substrate for the newly generated plasmin. As a control, we performed the assay in the absence of cells and confirmed the selectivity of the substrate, which was only degraded after activation of plasminogen by recombinant tPA (Figure 3A). We observed that the low amounts of plasminogen activators, tPA and uPA, endogenously produced by both untreated and adenosine A_2A_ agonist treated HDMVEC were not sufficient to activate plasminogen to plasmin (Figure 3B). To detect exclusively membrane-bound tPA-associated plasminogen activation it was necessary to incubate exogenous recombinant tPA for 30 min and then washed away before the addition of substrate and Glu-plasminogen. The lag time observed in plasmin generation by HDMVEC-surface bound tPA, about 20–30 min, is similar to that described for cerebral microvascular endothelial cells (Semov et al., 2005), but much longer than for pancreatic or breast cancer cells (Diaz et al., 2004; Sharma et al., 2010).

CGS-21680 pre-treatment produced an increase in membrane bound tPA-associated plasmin generation by HDMVEC (Figures 3B,C). Both preincubation with a specific antibody against annexin A2 (4 μg/ml) before binding of recombinant tPA, or the presence of the plasmin inhibitor 6-ACA (1 mM) in the final reaction, markedly inhibited plasmin generation and completely abolished the CGS-21680-mediated increase in cell associated-plasmin generation (Figure 3C).

### Plasmin and Annexin A2 Participates in the Proangiogenic Effect of an Adenosine A_2A_ Receptor Agonist

**FIGURE 4:**
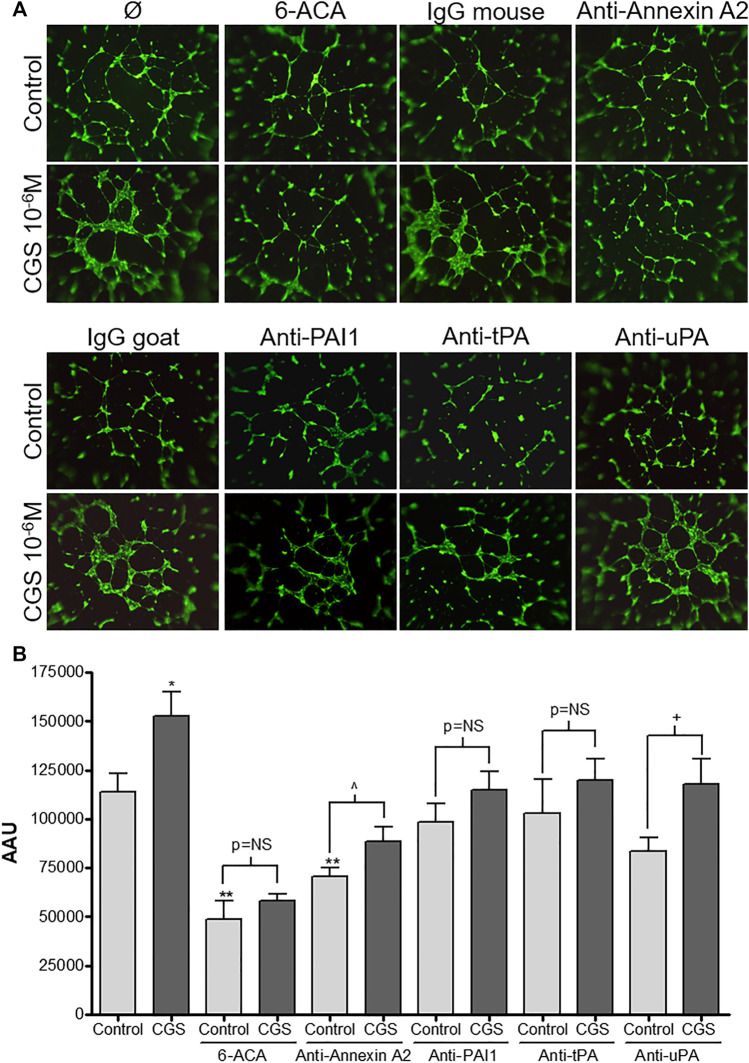
Microvascular endothelial cell network formation *in vitro*: HDMVEC (10^4^ cells, passage 4) were seeded in 200 µL of EGM-2MV onto 50 µL of polymerized Matrigel in 96 well plates. Cells were treated with either vehicle or CGS-216080 (10^−6^ M) in absence or presence of either the plasmin inhibitor 6-ACA (1 mM), blocking antibodies against annexin A2 (4 μg/ml), PAI-1, tPA or uPA (20 μg/ml) or their corresponding control mouse or goat IgG (20 μg/ml). For fluorescence detection of tubular network **(A)**, fluorophore/calcein AM (5 μM) was added after 18h incubation and image acquisition of tube formation was achieved by fluorescence microscopy (original magnification ×40). Total area of the cellular networks **(B)** was determined with SigmaScan Pro software and expressed as arbitrary area units (AAU). Results are presented as mean ± SEM (n = 3 or 4 in triplicate) *p < 0.05, **p < 0.01 vs. control; ^^^ *p* < 0.05 vs. anti-annexin A2 control; ^+^ *p*< 0.05 vs. anti-uPA control, ANOVA followed by Dunnett’s postest.

Once we established that adenosine A_2A_ receptor activation increased annexin A2 expression resulting in an increase in cell-bound tPA and thereby accelerated plasminogen activation, we evaluated the contribution of this system in another functional assay, the endothelial cell formation of a tubular network on a three-dimensional matrix. In a previous study, we demonstrated that A_2A_ receptor activation stimulates endothelial network formation in Matrigel and established the concentration dependent effect for the agonist CGS-21680 in this model (Desai et al., 2005). For the present study we selected the concentration of 1 μM, which had shown a maximal effect that could be abrogated by a selective A_2A_ antagonist (Desai et al., 2005). HDMVEC network formation was significantly inhibited by the plasmin inhibitor 6-ACA (1 mM) and a murine monoclonal antibody against annexin A2, but not a control murine antibody (Figure 4). In contrast, antibodies against tPA, uPA and PAI-1 did not affect basal network formation. Interestingly, adenosine A_2A_ receptor-mediated increases in network formation were blocked by 6-ACA and antibodies against PAI-1 and tPA but not uPA or annexin A2 (Figure 4).

## Discussion

We report here evidence for a novel mechanism by which adenosine receptors promote angiogenesis: stimulation of adenosine A_2A_ receptors promotes the expression of annexin A2 on the surface of endothelial cells, which binds tPA to the surface of the cells, providing for localized activation of plasminogen to plasmin. Moreover, adenosine A_2A_ receptor stimulation diminishes endothelial PAI-1 production as well. In the absence of tPA, adenosine receptor activation does not promote wound healing (Montesinos et al., 2015) and, when this mechanism is interrupted, adenosine A_2A_ receptor activation does not stimulate angiogenesis *in vitro*.

Endothelial cells derived from dermal microvasculature secreted small quantities of tPA into the culture medium, results which contrast with a prior report that human umbilical vein endothelial cells release much larger quantities of tPA (Speiser et al., 1987). Agents that increase intracellular cAMP levels (prostacyclin, isoproterenol, forskolin, cholera toxin) and cAMP analogs induce a dose and time-dependent acute release of tPA and Von Willebrand factor from HUVEC (Hegeman et al., 1998). Since signaling of adenosine A_2A_ receptor is supposed to occur through the activation of adenylate cyclase (Fredholm et al., 2011), we expected that selective doses of CGS21680 would induce tPA release from HDMVEC as well. However, we did not observe any appreciable change in tPA release, in agreement with an earlier report showing that cAMP analogs did not modify tPA secretion in non-stimulated HUVEC but potentiated the phorbol ester-induced secretion of tPA, and inhibited both basal and phorbol ester-increased PAI-1 secretion in HUVEC (Levin et al., 1989). Similarly, in this study we observed that adenosine A_2A_ receptor activation decreased the high levels of secreted PAI-1 in microvascular endothelial cells by decreasing both message expression and its subsequent release. This effect could be of clinical relevance, since high plasma levels of PAI-1 have been associated with vascular dysfunction and increased cardiovascular risks in several chronic pathological disorders such as hypertension, diabetes and metabolic syndrome (Naya et al., 2007; Lijnen, 2009; Sillen and Declerck, 2020).

The calcium-dependent phospholipid-binding protein annexin A2 is a proposed endothelial cell membrane co-receptor for plasminogen that can stimulate tPA–mediated plasminogen activation in the complete absence of fibrin (Hajjar et al., 1994) and plays an important role in angiogenesis by localizing plasmin activity on the endothelial cell surface (Ling et al., 2004). We then tried to explore a new hypothesis, that tPA could be indirectly involved in the promotion of wound revascularization by adenosine A_2A_ receptor activation through annexin A2. Selective doses of CGS21680 markedly augmented annexin A2 production by HDMVEC, which interacted with endogenously generated tPA. However, our results do not exclude the possibility that this interaction happened post-extraction, and it is not clear if the localization of annexin A2 is in the inner or outer leaflet of the plasma membrane of endothelial cells when its translocation was promoted by ionophore A23187 treatment.

Recently, controversy has emerged concerning the role of annexin A2 in binding plasminogen (Brodsky et al., 2001). The involvement of annexin A2 in the proteolytic activity of cell-bound tPA was confirmed since antibodies against the C-terminus of annexin A2 markedly reduced plasmin generation, although did not inhibited it completely, probably by blocking plasminogen binding to the lysine 307 at the C-terminus of annexin A2 (Liu and Hajjar, 2016). Similarly, the plasmin inhibitor 6-ACA which acts by blocking the plasminogen and plasmin binding site to lysine residues in either fibrin or the cell surface (Hatziapostolou et al., 2003), markedly inhibited cell-bound tPA-induced plasmin generation, although certain residual plasmin activity was still detectable, suggesting that in the cell microenvironment there are other proteases that could activate unbound plasminogen, since, as we observed in the absence of cells, the substrate is not degradable by inactive plasminogen.

The contribution of the different components of the plasminogen/plasmin system to the angiogenic process has been previously determined (Brodsky et al., 2001; Kruithof and Dunoyer-Geindre, 2014). The Matrigel^®^ *in vitro* assay had previously been used to study the proangiogenic effect of adenosine A_2A_ agonists (Desai et al., 2005) and the involvement of plasmin in the endothelial cell tubular network formation (Semov et al., 2005). Given that often incomplete lumen formation occurs (Stefansson et al., 2003), determination of total area constitutes a standardized method that facilitates comparisons among different conditions. Our results confirm that plasmin contributes to the angiogenic process, since the plasmin inhibitor 6-ACA suppressed endothelial cell tubular network formation, in a similar way as previously shown for cerebral microvascular endothelial cells (Semov et al., 2005). In contrast, antibodies against either uPA or tPA produced a small non-significant reduction in tube formation. Nevertheless, the anti-tPA antibody and the plasmin inhibitor prevented the stimulatory effect of the adenosine A_2A_ receptor agonist (characterized by formation of larger tubules), whereas the anti-uPA antibody had no effect, suggesting that plasminogen activation to plasmin by tPA participates in the proangiogenic effect of adenosine. Similarly, an antibody against PAI-1 did not block HDMVEC tubular formation on Matrigel, but prevented the stimulatory effect of CGS-21680. The role of PAI-1 in angiogenesis is controversial, while some authors have described PAI-1 as a limiting factor of *in vitro* angiogenesis assays (Stefansson et al., 2003), others have shown that PAI-1 expression is increased during tissue repair and remodeling to maintain the integrity of the fibrin matrix necessary for proper cellular migration (Isogai et al., 2001), probably through its interaction with vitronectin (Basu et al., 2009).

Tube formation by HDMVEC was greatly hindered by a neutralizing antibody against the C-terminus of annexin A2, which was modestly reversed by adenosine A_2A_ receptor activation, suggesting that the role of this protein in angiogenesis is not only due to its interaction with tPA. Annexin A2 belongs to the annexin family of proteins, which can bind membranes via negatively charged phospholipids and Ca^2+^ ions (Monastyrskaya et al., 2007). This could allow them to organize the interface between the cytoplasm and the cytoplasmic face of cellular membranes (Rescher and Gerke, 2004). The N-terminus of annexin A2 binds p11/S100A10 leading to the formation of a heterotetramer, resulting in a several fold increase in the Ca^2+^ sensitivity of membrane aggregation (Zibouche et al., 2008; Madureira et al., 2012). In addition, active cathepsin B, bound to the subunit p11/S100A10 of the annexin A2 heterotetramer (AIIt), partially localized to caveolae of human umbilical vein endothelial cells (HUVEC) in extracellular matrix degradation during tube formation (Cavallo-Medved et al., 2009). Blockade of annexin A2 function *in vivo*, by gene ablation or by a diet leading to hyper-homocysteinemia, leads to loss of endothelial cell surface plasmin generation, reducing endothelial cell migratory capacity that leads to angiogenic failure (Flood and Hajjar, 2011). Our results corroborate the more prominent role for Annexin A2 in plasminogen activation involved in angiogenesis, indistinctly of the heterotetramer formation with p11/S100A10, since blockade of the C-terminus markedly compromises both branching and tube length in the Matrigel^®^ assay. However, we cannot rule out the potentiating role of the two S100A10 molecules, which facing away from the membrane, create a platform for interaction with tPA in the extracellular space (Weisz and Uversky, 2020).

The results presented here corroborate the proangiogenic effect of adenosine A_2A_ receptor activation suggesting an indirect contribution of the tissue plasminogen activator through diminished release of its main inhibitor PAI-1 and increased expression of its cell surface receptor annexin A2.

## Data Availability

The raw data supporting the conclusions of this article will be made available by the authors, without undue reservation.
